# Higher pre-diagnostic serum syndecan-4 levels are associated with increased breast cancer risk: a case-cohort study

**DOI:** 10.1007/s10549-025-07786-4

**Published:** 2025-07-22

**Authors:** Endre Gabrielsen, Tom Wilsgaard, Hanne Frydenberg, Trygve Lofterød, Stig Manfred Dalen, Elin Mortensen, Marit D. Solbu, Hawa Nalwoga, Lars A. Akslen, Egil S. Blix, Hege S. Haugnes

**Affiliations:** 1 Department of Clinical Medicine, UiT – The Arctic University of Norway, 9037 Tromsø, Norway; 2 Department of Oncology, University Hospital of North Norway, 9038 Tromsø, Norway; 3 Department of Community Medicine, UiT – The Arctic University of Norway, Tromsø, Norway; 4 Department of Oncology, Oslo University Hospital, Oslo, Norway; 5 Department of Pathology, University Hospital of North Norway, Tromsø, Norway; 6 Department of Medical Biology, UiT – The Arctic University of Norway, Tromsø, Norway; 7 Section of Nephrology, University Hospital of North Norway, Tromsø, Norway; 8 Centre for Cancer Biomarkers (CCBIO), Department of Clinical Medicine, University of Bergen, Bergen, Norway; 9 Department of Pathology, Makere University College of Health Sciences, Kampala, Uganda; 10 Department of Oncology and Pathology, Karolinska Institutet, Stockholm, Sweden

**Keywords:** Syndecan 1, Syndecan 4, Breast cancer, Hormone receptor-positive, Risk factors, Case-cohort study

## Abstract

**Purpose:**

Syndecans are transmembrane proteins involved in inflammation and signaling pathways. Their potential role as pre-diagnostic biomarkers for breast cancer risk remains unexplored. This study aimed to investigate whether pre-diagnostic serum syndecan levels are associated with breast cancer risk in a population-based cohort.

**Methods:**

We conducted a case-cohort study nested within the Tromsø Study (Norway), including women who participated in the fifth survey (2001). Women with incident breast cancer (cases, n = 158) through 2022 were identified, with a random sub-cohort of 708 women. Serum levels of syndecan-1 (SDC1) and syndecan-4 (SDC4) were measured using ELISA on frozen serum samples obtained in 2001. All participants were stratified into quartiles (Q1–Q4) based on pre-diagnostic levels. Cox proportional hazards regression models assessed associations between serum syndecan levels and breast cancer risk.

**Results:**

The median age at diagnosis was 69 years for cases, and 83.3% of tumors were hormone receptor-positive (HR +). Women with higher serum SDC4 (Q2–Q4) levels had approximately a twofold increased risk of breast cancer compared to women in Q1. We observed a nearly threefold increased risk for the HR + subtype. In postmenopausal women, HRs for HR + breast cancer in Q2, Q3, and Q4 were 3.81 (95% CI: 1.57–9.23), 3.43 (95% CI: 1.41–8.40), and 3.54 (95% CI: 1.45–8.65), respectively, all relative to Q1 of SDC4. No associations were observed between SDC1 levels and breast cancer risk.

**Conclusions:**

Our results suggest that SDC4 may play a role in the initiation and early progression of breast cancer.

**Supplementary Information:**

The online version contains supplementary material available at 10.1007/s10549-025-07786-4.

## Background

Breast cancer is the most prevalent malignancy among women globally, accounting for about 30% of all cancer cases [1]. Increasingly, breast cancer has been recognized as a heterogeneous disease, with tumor receptor status playing a critical role in determining treatment choices and prognosis. Despite significant advancements in therapy, breast cancer remains a major cause of cancer-related mortality worldwide [1]. Early detection may improve survival outcomes, reduce the need for aggressive, long-term treatments, and minimize treatment-related adverse effects [1]. Identifying reliable biomarkers is crucial for increasing early detection rates and improving personalized breast cancer treatments.

Inflammation has been recognized as a critical factor in the initiation and progression of various malignancies [2], and elevated serum inflammatory biomarkers have been associated with an increased risk of breast cancer [3, 4]. A potential marker of inflammation is the shedding of syndecans. This process can accelerate under normal physiological conditions such as wound healing [5, 6], and in pathological conditions, including sepsis and cancer [7, 8].

The syndecans are a family of cell surface heparan sulfate proteoglycans comprising four members: syndecan-1 (SDC1), syndecan-2, syndecan-3, and syndecan-4 (SDC4). These proteoglycans play multifaceted roles in cancer biology, influencing key processes such as cell proliferation, migration, invasion, and angiogenesis [7, 9–11]. Most human cells express at least one member of the syndecans [8, 12]. SDC1 is highly expressed in epithelial tissue, whereas SDC4 is more ubiquitously expressed across different tissues [7, 9, 12]. Structurally, syndecans consist of a cytoplasmic domain, a transmembrane region, and an extracellular domain, the latter containing glycosaminoglycan (GAG) chains composed of either heparan-sulfate (HS) or chondroitin sulfate [7]. The negatively charged GAG chains enable syndecans to interact with various ligands, including growth factors and cytokines, thereby regulating diverse cellular functions [7].

Proteolytic cleavage at the juxtamembrane region results in the release of soluble ectodomains into the bloodstream. These soluble syndecans can act as autocrine or paracrine signaling molecules or decoy receptors by competing with membrane-bound syndecans for ligand binding [6, 9]. Syndecan shedding has been proposed as a biomarker of inflammation-associated processes in cancer [13].

Elevated levels of soluble syndecans, particularly SDC1 and SDC4, have been correlated with adverse outcomes in various cancer types [9, 10, 14]. Of note, most existing studies have investigated syndecan shedding in a post-diagnostic setting, leaving a gap in knowledge regarding its potential role in cancer initiation and early progression. Thus, this study primarily aimed to investigate whether pre-diagnostic serum levels of SDC1 and SDC4 were associated with breast cancer risk in a prospective population-based cohort. Additionally, we explored whether pre-diagnostic syndecan levels correlate with breast cancer-specific survival (BCSS) and overall survival (OS) among women diagnosed with breast cancer within the same cohort.

## Methods

### Study design and participants

**Fig. 1:**
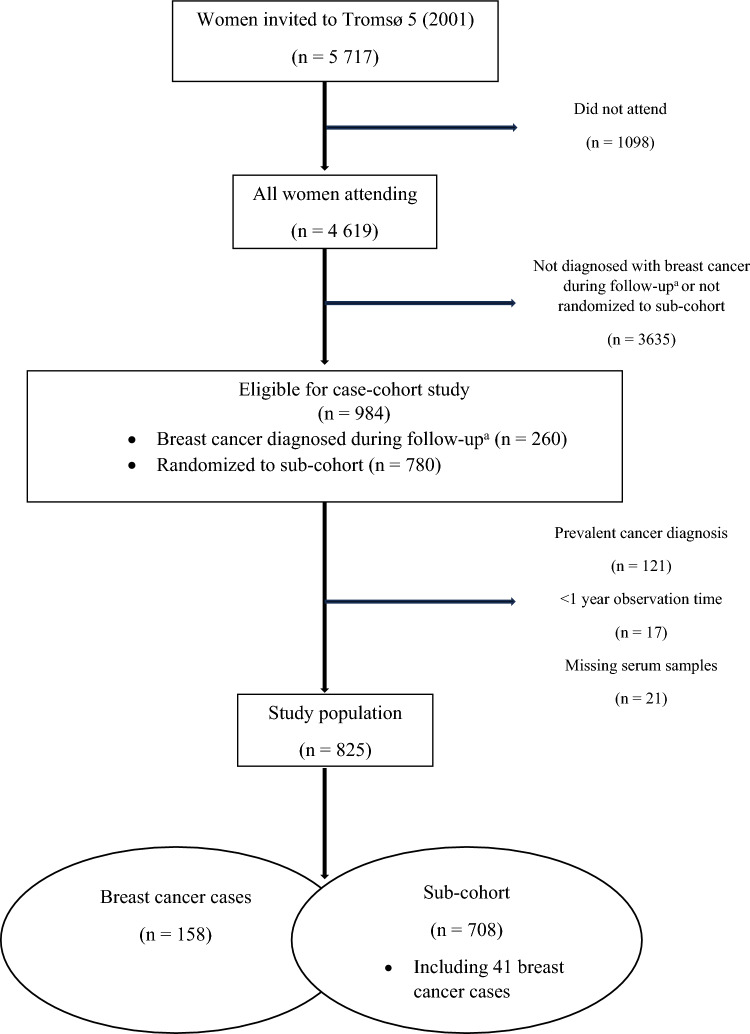
Women participating in the case-cohort study nested in Tromsø5 (2001). ^a^ December 31, 2022

Women who participated in the Tromsø5 Study (2001) [15] were eligible for inclusion in this study, which is a sub-study of the Energy Balance and Breast Cancer Aspects throughout life (EBBA-Life) study [16, 17]. The Tromsø Study is an ongoing prospective, population-based study in Tromsø, Northern Norway [15]. For this project, we carried out a case-cohort study nested within Tromsø5. Overall, 5717 women were invited to participate via personal invitation, of whom 4,619 women attended (80.8%) [15]. We identified all women (n = 260) who participated in Tromsø 5 and were diagnosed with breast cancer by the end of follow-up (December 31, 2022) (Fig. 1). A random sample three times the size of the case group (n = 780) was selected to form the sub-cohort. Women with any prevalent cancer diagnosis at the time of attendance (n = 121) or who had insufficient serum samples (n = 21) were excluded. To minimize potential bias from severe undiagnosed illness, we also excluded individuals who were diagnosed with any type of cancer, emigrated, or died within the first year of follow-up (n = 17). A total of 825 women constituted our study population, of whom 158 were diagnosed with breast cancer by the end of follow-up (cases). The sub-cohort consisted of 708 women, including 41 women who developed breast cancer during follow-up.

### Questionnaires, clinical assessments, and blood sampling

Sociodemographic (education), reproductive (age at menarche, number of children, menopausal status), lifestyle (smoking, physical activity, alcohol use), and medical history (antihypertensive drugs, hormone replacement therapy (HRT)) were collected via self-administered questionnaires completed before clinical examination [18]. Non-responders received one reminder.

Physical activity was categorized as active (≥ 3 h of light or ≥ 1 h of strenuous physical activity in leisure time per week) or inactive. Alcohol use was also dichotomized as high (≥ 2 times/month) or low (< 2 times a month). Women were categorized as postmenopausal if they self-reported menopause, had elevated follicle-stimulating hormone (FSH) levels (≥ 40 IU/L)[19], and/or current age ≥ 55 years [20].

Height and weight were measured using a regularly calibrated electronic scale. Body mass index (BMI) was calculated (kg/m^2^) [21]. Blood pressure was measured three times at rest with the average of the last two measurements used. Hypertension was defined as systolic blood pressure ≥ 140 mmHg, diastolic blood pressure ≥ 90 mmHg, or current antihypertensive medication use.

Non-fasting blood samples were collected at the study entry. Biomarker concentrations were reported as follows: Cholesterol, triglycerides, and glucose in millimoles per liter (mmol/L), C-reactive protein (CRP) (mg/L), white blood cell (WBC) count (L), ferritin, and fibrinogen in grams per liter (g/L). All analyses, except for SDC1 and SDC4, were conducted at the ISO-accredited Department of Laboratory Medicine, University Hospital of Northern Norway [21]. Details of laboratory analysis have been described previously [15, 18].

### Measurements of SDC1 and SDC4

Serum samples collected in 2001 were stored at − 20 °C until analysis. Before measurements, samples were thawed overnight in a refrigerator, vortexed, and centrifuged. Serum levels of human SDC1 (Diaclone, 950.640.192) and SDC4 (IBL, JP 27188) were quantified by commercial ELISA kits. Analysts were blinded to clinical data. Additional details are provided in the Supplementary Material.

### Identification of breast cancer cases and breast tumor characteristics

Breast cancer cases diagnosed during follow-up were identified using the Cancer Registry of Norway and the unique, 11-digit national identification number. Data on emigration and mortality were obtained via linkage to the National Population Registry and the Cause of Death Registry. Breast cancer deaths were identified using ICD-10 codes C50-C50.9 from the underlying cause of death in the Cause of Death Registry.

Detailed histological information on breast cancer cases was retrieved from medical records by trained physicians (EG, SMD, TL, HF). To ensure consistency in tumor characteristics over time (2001–2022), tumor tissue from women diagnosed before 2013 (n = 90) was re-examined [16]. All tumor samples had been fixed in 4% buffered formaldehyde before processing and embedded in paraffin. Pathologists (EM, HN, LAA) selected representative tumor regions from samples to obtain cores for constructing a tissue microarray (TMA). TMA blocks were constructed and analyzed (by HN and LAA) at the Centre for Cancer Biomarkers, CCBIO, Section for Pathology, University of Bergen. Immunohistochemistry (IHC) determined estrogen receptor (ER), progesterone receptor (PR), and human epidermal growth factor receptor 2 (HER2) status. Tumors were ER-positive if 1% ≥ of nuclei stained positive, PR-positive if ≥ 10% nuclei stained positive, and HER2-positive if ≥ 10% of cell membranes showed 3 + staining. HER2 silver in situ hybridization (SISH) was performed for HER2 IHC2 + cases and considered positive if the HER2/Chromosome 17 ratio was ≥ 2.0 [22]. Cases diagnosed after 2012 (n = 68) were analyzed using IHC for hormone receptor status and fluorescence ISH for HER2. Discrepancies between TMA analyses and medical journals were resolved by prioritizing histological reports. Cases were considered hormone receptor-positive (HR +) if either ER or PR was positive and hormone receptor-negative (HR-) if both ER and PR were negative.

Histology was categorized as invasive carcinoma of no special type (ICNST), invasive lobular carcinoma, or other types. Tumor grade was assigned using Nottingham criteria [23]. Tumor size was measured in millimeters and classified by TNM staging. Lymph node metastases were recorded as yes/no, and overall cancer stage (I-IV) was assigned using TNM 8th edition [24].

### Statistical methods

Descriptive characteristics are presented as median [interquartile range (IQR)] for continuous variables and number (percentage) for categorical variables.

Multivariable Cox proportional hazards models, with follow-up time as the timescale, were used to estimate hazard ratios (HR) and 95% confidence intervals (CI) for the association between pre-diagnostic serum levels of SDC1 and SDC4, breast cancer risk, subtype-specific breast cancer risk, BCSS, and OS. Follow-up time for breast cancer risk and subtype-specific breast cancer risk was calculated from baseline (date of participation in Tromsø5) to the earliest of breast cancer diagnosis, death, emigration, or end of follow-up (December 31, 2022). For BCSS and OS, only women with a breast cancer diagnosis (n = 140 for SDC1, n = 158 for SDC4) were included. Follow-up time was calculated from breast cancer diagnosis to the earliest of death, emigration, or end of follow-up.

SDC1 and SDC4 were analyzed as continuous variables (HR per standard deviation (SD) increase) and categorical variables (quartiles (Q) based on sub-cohort distribution). Quartile cut-offs were:

- **SDC1**: Q1 (≤ 62.67 ng/mL), Q2 (62.68–87.60 ng/mL), Q3 (87.61–131.99 ng/mL), Q4 (≥ 132.00 ng/mL).
- **SDC4**: Q1 (≤ 16.08 ng/mL), Q2 (16.09–19.60 ng/mL), Q3 (19.61–23.69 ng/mL), Q4 (≥ 23.70 ng/mL).

Models were adjusted for baseline age (continuous) and additional covariates based on proposed biological mechanisms: alcohol consumption (high/low), physically active (yes/no), number of children (continuous), and BMI (continuous). Other variables (hypertension, smoking, age at menarche, estrogen use, CRP, and WBC did not influence results and were excluded from final models. To assess menopausal status interactions, we included cross-product terms between each indicator variable of SDC4 quartile and menopause status in a separate model. Interaction was tested using a likelihood ratio test(chi-squared statistic, 3 degrees of freedom). Subgroup analysis was performed by menopausal status (postmenopausal yes/no).

The proportional hazard assumption was evaluated using graphical methods and residual-based tests. Cumulative incidence curves for SDC1 and SDC4 quartiles were generated using the Kaplan–Meier method. Forest plots illustrated age-adjusted HRs with 95% CIs for selected blood biomarkers and breast cancer risk. Sensitivity analyses using alternative cut-offs (tertiles and quintiles) and subgroup analyses (e.g. alcohol consumption, CRP, and WBC) confirmed the robustness of results.

All statistical analyses were conducted using Stata version 18 (StataCorp, College Station, TX, USA).

## Results

### Characteristics

At study entry, the median age was 60 years [IQR: 45–66] for women later diagnosed with breast cancer and 61 years [IQR: 45–69] for women in the sub-cohort (Table 1). The majority of women were postmenopausal (74.7% in the cases group and 74.0% in the sub-cohort). Median follow-up time from baseline was shorter for women diagnosed with incident breast cancer (10 years [IQR: 5–15]) than for the sub-cohort (20 years [IQR: 16–21]). Pre-diagnostic median SDC1 levels were 87.8 ng/mL [IQR: 65.6–126.0] for breast cancer cases and 87.6 ng/mL [IQR: 62.7–132.0] in the sub-cohort, while pre-diagnostic SDC4 levels were 20.5 ng/mL [IQR: 17.6–24.9] and 19.5 ng/mL [IQR: 16.1–23.8], respectively.

**Table 1:** Baseline characteristics of women diagnosed with breast cancer and the sub-cohort

Characteristic	Breast cancer cases (n = 158)^a^ median [IQR]/n (%)	Sub-cohort (n = 708)^a^ median [IQR]/n (%)
Age, years	60 [45–66]	61 [45–69]
Education, years	10 [7–13]	10 [7–13]
Clinical measurements		
BMI, kg/m^2^	25.9 [23.7–29.6]	25.8 [23.1–29.2]
Hypertension^b^	81 (51.6)	356 (50.4)
Lifestyle variables		
Current smoking	51 (32.3)	202 (28.7)
Physically active^c^	127 (84.7)	525 (80.5)
Alcohol use^d^	90 (58.4)	351 (50.9)
Estrogen-related variables		
Menarche age, years	13 [12–14]	13 [12–14]
Number of children	2 [1–3]	2 [2, 3]
Postmenopausal status	118 (74.7)	524 (74.0)
Current or former use of estrogen^e^	46 (31.9)	167 (27.5)
Blood samples		
Cholesterol, mmol/L	6.15 [5.23–6.93]	6.29 [5.38–7.09]
Triglycerides, mmol/L	1.25 [0.92–1.80]	1.24 [0.91–1.81]
Glucose, mmol/L	5.16 [4.75–5.65]	5.13 [4.76–5.60]
CRP, mg/L	1.79 [0.89–3.53]	1.63 [0.81–3.00]
WBC, 10^9^/L	6.15 [5.30–7.20]	6.10 [5.20–7.20]
Fibrinogen, g/L	3.40 [2.80–3.70]	3.20 [2.70–3.60]
Ferritin, g/L	57.0 [30.5–86.9]	59.0 [32.0–91.5]
Syndecan-1, ng/mL	87.8 [65.6–126]	87.6 [62.7–132]
Syndecan-4, ng/mL	20.5 [17.6–24.9]	19.6 [16.1–23.8]
Characteristics of breast cancer cases		
Age at diagnosis, years	69 [59–78]	
Histologic type		
Invasive carcinoma NST	123 (80.9)	
Invasive lobular carcinoma	22 (14.5)	
Other	7 (4.6)	
Histologic grade		
1	45 (30.0)	
2	78 (52.0)	
3	27 (18.0)	
T-stage		
1	83 (56.5)	
2	42 (28.6)	
3	11 (7.5)	
4	11 (7.5)	
Lymph node metastasis		
Yes	49 (33.8)	
Stage		
1	65 (42.8)	
2	55 (36.2)	
3	21 (13.8)	
4	11 (7.2)	
Receptor status		
ER +	124 (78.5)	
PR +	92 (58.2)	
HER2 +	29 (18.4)	
TNBC	15 (9.5)	
HR +		
Yes	125 (83.3)	

^a^Continuous variables are presented as median [IQR], and categorical variables are presented as n (%). Numbers may vary due to missing data

^b^Mean systolic pressure ≥ 140 mmHg, mean diastolic pressure ≥ 90 mm/hg, or use of antihypertensive medication

^c^Engaged in at least three hours of light physical activity in leisure time per week or at least one hour of strenuous physical activity in leisure time per week over the past year

^d^Use of alcohol at least twice per month

^e^Current or former estrogen use (tablets or patches)

*n* number of cases, *IQR* interquartile range, *BMI* Body mass index, *CRP* C-reactive protein, *WBC* white blood cells, *NST* no special type, *ER* +  Estrogen receptor positive, *PR* +  Progesterone receptor-positive, *HR* +  hormone receptor-positive, *HER2* +  human epidermal growth factor receptor 2 positive, *TNBC* triple-negative breast cancer

Among breast cancer cases, the median age at diagnosis was 69 years [IQR: 59–78], 80.9% were diagnosed with ICNST, and 79.0% were classified as stage I or II. Based on tumor receptor status, 83.3% were classified as HR + , 18.4% as HER2 + , and 9.5% as triple-negative breast cancer (TNBC).

### SDC1, SDC4, and overall breast cancer risk

**Fig. 2:**
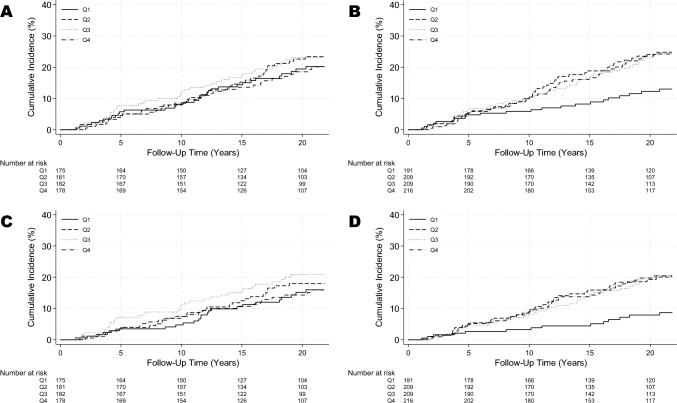
Cumulative incidence of breast cancer by quartiles (Q) of pre-diagnostic serum syndecan levels during follow-up. **A** Syndecan-1, overall breast cancer incidence; **B** syndecan-4, overall breast cancer incidence; **C** syndecan-1, hormone receptor-positive breast cancer incidence; **D** syndecan-4, hormone receptor-positive breast cancer incidence

No associations were observed between pre-diagnostic SDC1 levels and breast cancer (Fig. 2A, Table 2). The cumulative 15-year incidence of breast cancer was 8.2% (95% CI: 0.05–13.3) for women with pre-diagnostic SDC4 levels in Q1, compared to 18.8% (95% CI: 13.9–25.2) in Q2, 16.6% (95% CI: 12.0–22.7) in Q3, and 16.6% (95% CI: 12.2–22.5) in Q4 (Fig. 2B).

**Table 2:** Age- and multivariable-adjusted hazard ratios for pre-diagnostic syndecan-1, syndecan-4, and breast cancer risk

	Cases/sub-cohort (n)	HR^a^ (95% CI)	Cases/sub-cohort (n)	HR^b^ (95% CI)
Syndecan-1				
Continuous, per 1 SD^c^ increase	140/613	0.87 (0.68–1.11)	131/544	0.91 (0.73–1.13)
Quartiles				
Q1: ≤ 62.67 ng/mL	32/154	1.00 (Reference)	29/140	1.00 (Reference)
Q2: 62.67–87.60 ng/mL	38/153	1.13 (0.70–1.81)	34/138	1.20 (0.73–1.97)
Q3: 87.61–132.00 ng/mL	38/153	1.16 (0.73–1.86)	36/135	1.35 (0.82–2.21)
Q4: ≥ 132.00 ng/mL	32/153	0.96 (0.59–1.57)	32/131	1.22 (0.73–2.04)
Syndecan-4				
Continuous, per 1 SD^d^ increase	**158/708**	**1.23 (1.06–1.43)**	**147/626**	**1.25 (1.07–1.46)**
Quartiles				
Q1: ≤ 16.08 ng/mL	22/177	1.00 (Reference)	19/161	1.00 (Reference)
Q2: 16.08–19.60 ng/mL	45/177	**1.98 (1.19–3.30)**	42/158	**2.23 (1.29–3.84)**
Q3: 19.61–23.70 ng/mL	44/177	**1.92 (1.15–3.20)**	43/150	**2.20 (1.28–3.79)**
Q4: ≥ 23.70 ng/mL	47/177	**1.95 (1.17–3.23)**	43/157	**2.11 (1.23–3.63)**

^a^Age-adjusted (continuous)

^b^Adjusted for age (continuous), alcohol consumption (dichotomized as high/low), physically active (dichotomized as yes/no), number of children (continuous), and BMI (continuous)

^c^SD = 197.81 ng/ml

^d^SD = 5.75 ng/ml

Statistically significant (p-value < 0.05) hazard ratios are marked in bold letters

*HR* hazard ratio, *CI* confidence interval, *SD* standard deviation, *n* number of participants, *Q* quartile, *BMI* body mass index in kilogram per square meter

Median age at diagnosis according to SDC4 levels was 68.6 years [IQR: 56.5–78.8] in Q1, 68.7 years [IQR: 61.5–76.9] in Q2, 67.2 years [IQR: 57.2–78.5] in Q3, and 69.1 years [IQR: 62.0–77.0] in Q4. In multivariable-adjusted models, HRs for incident breast cancer were 2.23 (95% CI: 1.29–3.84) in Q2, 2.20 (95% CI: 1.28–3.79) in Q3, and 2.11 (95% CI: 1.23–3.63) in Q4, all relative to Q1 of SDC4 (Table 2).

**Fig. 3:**
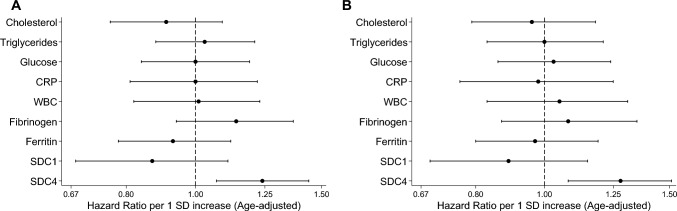
Age-adjusted hazard ratios for pre-diagnostic inflammatory biomarkers and breast cancer risk per 1-SD increase. **A** overall breast cancer risk; **B** hormone receptor-positive breast cancer risk. *CRP* C-reactive protein, *WBC* white blood cells, *SDC1* Syndecan-1, *SDC4* Syndecan-4, *SD* standard deviation

No associations were observed between other available pre-diagnostic inflammatory serum biomarkers (cholesterol, triglycerides, glucose, CRP, WBC, ferritin) and breast cancer risk (Fig. 3A).

### SDC1, SDC4, HR + breast cancer risk, and survival

We observed no associations between pre-diagnostic SDC1 levels and HR + breast cancer incidence (Fig. 2C). For SDC4, the cumulative 15-year incidence of HR + breast cancer was 4.4% (95% CI: 2.2–8.6) in Q1, 15.9% (95% CI: 11.4–22.9) in Q2, 13.9% (95% CI: 9.7–19.8) in Q3, and 14.3% (95% CI: 10.1–19.9) in Q4 (Fig. 2D). Compared with Q1 of serum SDC4 levels, multivariable-adjusted HRs for HR + breast cancer were 2.86 (95% CI: 1.47–5.30), 2.90 (95% CI: 1.50–5.59), and 2.79 (95% CI: 1.44–5.38) for Q2, Q3, and Q4, respectively (Table 3). We tested the interaction between SDC4-quartiles and menopause status in the multivariable-adjusted Cox model, finding no significant interaction (χ^2^(3) = 1.83, p = 0.61). However, subgroup analysis revealed stronger associations in postmenopausal women, and HRs in Q2, Q3, and Q4 were 3.81 (95% CI: 1.57–9.23), 3.43 (95% CI: 1.41–8.40), and 3.54 (95% CI 1.45–8.65), respectively, all relative to Q1 of SDC4. We also assessed pre-diagnostic SDC1 and SDC4 levels in HR- breast cancer (n = 25), finding no significant associations in multivariable-adjusted models (data not shown).

**Table 3:** Age- and multivariable-adjusted hazard ratios for pre-diagnostic syndecan-4 and hormone receptor-positive breast cancer risk

	Cases/sub-cohort (n)	HR^a^ (95% CI)	Cases/sub-cohort (n)	HR^b^ (95% CI)
Overall				
Continuous, per 1 SD^c^ increase	125/708	**1.28 (1.08–1.51)**	119/626	**1.29 (1.09–1.53)**
Quartiles				
Q1: ≤ 16.08 ng/mL	14/177	1.00 (Reference)	13/161	1.00 (Reference)
Q2: 16.08–19.60 ng/mL	36/177	**2.52 (1.35–4.67)**	34/158	**2.86 (1.47–5.30)**
Q3: 19.61–23.70 ng/mL	36/177	**2.49 (1.34–4.61)**	36/150	**2.90 (1.50–5.59)**
Q4: ≥ 23.70 ng/mL	39/177	**2.55 (1.38–4.70)**	36/157	**2.79 (1.44–5.38)**
Premenopausal				
Continuous, per 1 SD^c^ increase	35/184	1.22 (0.87–1.70)	35/179	1.29 (0.91–1.83)
Quartiles				
Q1: ≤ 16.08 ng/mL	7/59	1.00 (Reference)	7/58	1.00 (Reference)
Q2: 16.08–19.60 ng/mL	7/40	1.47 (0.52–4.20)	7/39	1.39 (0.49–4.00)
Q3: 19.61–23.70 ng/mL	11/41	2.07 (0.79–5.42)	11/40	2.01 (0.76–5.30)
Q4: ≥ 23.70 ng/mL	10/44	1.73 (0.66–4.54)	10/42	1.89 (0.71–5.00)
Postmenopausal				
Continuous, per 1 SD^c^ increase	90/524	**1.30 (1.07–1.58)**	84/447	**1.32 (1.08–1.61)**
Quartiles				
Q1: ≤ 16.08 ng/mL	7/118	1.00 (Reference)	6/103	1.00 (Reference)
Q2: 16.08–19.60 ng/mL	29/137	**3.42 (1.50–7.80)**	27/119	**3.81 (1.57–9.23)**
Q3: 19.61–23.70 ng/mL	25/136	**2.98 (1.29–6.90)**	25/110	**3.43 (1.41–8.40)**
Q4: ≥ 23.70 ng/mL	29/133	**3.35 (1.47–7.64)**	26/115	**3.54 (1.45–8.65)**

^a^Age-adjusted (continuous)

^b^Adjusted for age (continuous), alcohol consumption (dichotomized as high/low), physically active (dichotomized as yes/no), number of children (continuous) and BMI (continuous)

^c^SD = 5.75 ng/ml

Statistically significant (p value < 0.05) hazard ratios are marked in bold letters

*HR* hazard ratio, *CI* confidence interval, *HR* +  hormone receptor-positive, *SD* standard deviation, *n* number of participants, *BMI* body mass index in kilogram per square meter

For other pre-diagnostic inflammatory biomarkers, we found no associations with HR + breast cancer risk (Fig. 3B).

Among women diagnosed with breast cancer, pre-diagnostic levels of SDC1 and SDC4 were not associated with BCSS or OS in age-adjusted Cox proportional hazards models (Supplementary Table S1).

## Discussion

In the present case-cohort study of women attending a prospective population-based study, higher levels of pre-diagnostic SDC4 were associated with an increased breast cancer risk. For the HR + subtype, women in the lowest SDC4 quartile had nearly one-third of the risk compared to higher quartiles. This pattern suggests a potential threshold effect, where SDC4 levels below a certain point may confer protection, while elevated levels contribute to increased breast cancer risk. We observed no associations between pre-diagnostic SDC1 levels and breast cancer risk.

HR + breast cancer is more likely to be influenced by risk factors related to lifestyle and reproductive factors compared to other subtypes [25, 26]. Therefore, it is essential to distinguish between subtypes when examining risk factors for breast cancer, as the risk of some subtypes can be, to some extent, modifiable [27].

A previous study on tumor tissue also reported the association between SDC4 expression and HR + breast cancer [28]. However, their findings contrasted with those of an earlier study [29], possibly due to differences in antigen retrieval methods and the antibodies used. Whether serum SDC4 levels reflect syndecan shedding from HR + breast tumors or serve as markers of systemic inflammation influencing cancer risk remains unclear and, to our knowledge, unexplored. The precise cellular sources of serum SDC4 are not entirely determined, but endothelial cells are hypothesized as significant contributors [30–32].

The association between pre-diagnostic serum SDC4 levels and HR + breast cancer risk was notably stronger in postmenopausal women compared to premenopausal women. This disparity may be related to the influence of sex hormones on serum SDC4 levels, which could mask or modify its effect in premenopausal women. Previous research has shown that SDC4 gene expression, but not protein expression, fluctuates in normal breast tissue across the menstrual cycle, potentially reflecting hormonal regulation [33]. Similarly, a study on endometrial tissue demonstrated that specific matrix metalloproteinases (MMPs), including MMP-9, are predominantly expressed during the menstrual phase, suggesting a hormonally driven mechanism that may also affect SDC4 dynamics [34].

Apart from serum syndecans, our study had access to various inflammatory biomarkers, including WBC, CRP, fibrinogen, and ferritin. Only SDC4 demonstrated a significant association with overall and HR + breast cancer risk. This specificity sets SDC4 apart from other markers of inflammation. The unique association of SDC4 may reflect its distinct role in cellular processes, including extracellular matrix remodeling and growth factor signaling, potentially linked to HR + tumor biology [35].

Our study has several strengths. Primarily, it is nested within the prospective population-based Tromsø Study, and the attendance proportion exceeds 80% of women invited to Tromsø5 [15]. Additional strengths include a standardized protocol for data collection, near-complete identification of breast cancer cases through mandatory reporting to the Cancer Registry of Norway, rigorous validation of questionnaires, and comprehensive tracking of deaths and emigration through the Cause of Death Registry and the National Population Registry. These features enhance the representativeness of our findings to the source population and minimize the potential impact of loss to follow-up. The case-cohort design allowed us to explore the association between pre-diagnostic SDC1 and SDC4 and breast cancer while preserving statistical power. Among breast cancer cases, clinical and histological data were carefully obtained, and most of the tumors were reanalyzed using TMA, enabling close to complete tumor characterization and comparison across invasive breast cancer cases diagnosed at various time points. In addition, laboratory analyses of serum SDC1 and SDC4 were conducted using validated ELISA methods. Finally, a follow-up time exceeding 20 years strengthens long-term risk assessment.

However, several limitations should also be acknowledged. Our measurements did not differentiate shedding sites or modifications of HS-GAG chains, which may reflect distinct physiological processes [36]. For a subset of participants in our study (n = 80), we used SDC4 levels measured in 2016 as part of an earlier study [37]. Additionally, the impact of diurnal variation and influences beyond inflammation on serum syndecan levels remains, to our knowledge, unknown. We have only one observation point, preventing an evaluation of temporal dynamics. The absence of a linear trend for SDC4 and breast cancer risk and variability in SDC1 measurements may also have influenced our findings. In addition, the relatively small case sample size (n = 158) may have limited statistical power for subgroup analyses. Finally, samples stored for over 20 years at − 20 °C may have been subject to degradation, potentially affecting biomarker levels.

## Conclusion

In summary, higher pre-diagnostic serum SDC4 levels were associated with an increased incidence of breast cancer, particularly among postmenopausal women with the HR + subtype. Conversely, women in the lowest SDC4 quartile exhibited a protective effect, suggesting a threshold below which SDC4 may reduce risk. These findings, potentially influenced by cohort-specific factors, require validation in larger, independent cohorts. To our knowledge, this study is the first to assess pre-diagnostic SDC1 and SDC4 levels and breast cancer risk, offering novel insights into the knowledge of pre-diagnostic levels of SDC4 as a potential pathogenesis factor and as a potential biomarker of inflammation-associated processes in breast cancer development.

## Supplementary Information

Below is the link to the electronic supplementary material.

Supplementary file1 (DOCX 21 KB)

## Data Availability

The data supporting this study are not publicly available due to ethical and privacy restrictions imposed by the Data Protection Officer and the Regional Committee for Medical and Health Research Ethics. Access to anonymized data may be requested through the Tromsø Study data repository, subject to approval.

## Abbreviations

SDC1: Syndecan-1
SDC4: Syndecan-4
GAG: Glycosaminoglycan
HS: Heparan-sulfate
EBBA-Life: Energy balance and breast cancer aspects throughout life
HRT: Hormone replacement therapy
FSH: Follicle-stimulating hormone
CRP: C-reactive protein
WBC: White blood cell
ELISA: Enzyme-linked immunosorbent assay
CV: Coefficient of variation
EG: Endre Gabrielsen
TL: Trygve Lofterød
HF: Hanne Frydenberg
SMD: Stig Manfred Dalen
EM: Elin Mortensen
HN: Hawa Nalwoga
LAA: Lars A. Akslen
TMA: Tissue microarray
IHC: Immunohistochemistry
ER: Estrogen receptor
PR: Progesterone receptor
HER2: Human epidermal growth factor receptor
ISH: In situ hybridization
HR +: Hormone receptor-positive
ICNST: Invasive carcinoma of no special type
IQR: Interquartile range
HR: Hazard ratio
CI: Confidence intervals
Q: Quartiles
SD: Standard deviation
REK: Regional committee for medical and health research ethics
TNBC: Triple negative breast cancer
MMP: Matrix metalloproteinase
