# Supplementary material for: Higher pre-diagnostic serum syndecan-4 levels are associated with increased breast cancer risk: a case-cohort study
Source: Breast Cancer Res Treat. 2025 Jul 22;213(3):365–75. doi: 10.1007/s10549-025-07786-4 (PMC12397127; doi:10.1007/s10549-025-07786-4)
Supplement: Supplementary file 1 — Supplementary file1 (DOCX 21 KB) [file 10549_2025_7786_MOESM1_ESM.docx]

**Supplementary Material**

Syndecan-1 (SDC1) and syndecan-4 (SDC4) levels for a subset of participants (n=108) were previously measured in 2016 as part of an earlier study [1]. These data were combined with new measurements from the remaining cohort (n=717), conducted in 2024 using identical ELISA kits from the same manufacturers. All ELISA plates used in 2016 and 2024 were from single batches. SDC1 measurements from 2016 and 2024 exhibited right-skewed distributions and high variability. Additionally, the mean for the 2016 measurements of SDC1 was 52.1 ng/mL (standard deviation (SD) = 33.5), while the mean for the 2024 measurements was substantially higher at 137.1 ng/mL (SD = 204.1). Among the 28 patients with paired measurements, the correlation between the two time points was 0.79. Due to inconsistencies between the 2016 and 2024 measurements, we used only the 2024 data for SDC1. The interassay coefficient of variation (CV) for SDC1 was 13.5%. Less than 10% of samples fell outside the detection range and were diluted to obtain absolute values.

In contrast, SDC4 measurements exhibited normal distributions with consistent values across both time points. For the samples analyzed in 2016, the mean was 19.2 ng/ml (SD = 5.8); for those surveyed in 2024, the mean was 20.2 ng/ml (SD = 5.8). All samples analyzed in 2024 were within the limits of detection. Using sample controls, interassay CV was 6.1% across all plates. Samples analyzed in 2016 were within the detection limits, and the interassay CV was 6.7% [1]. For patients with measurements available from both 2016 and 2024, the 2024 data were used.

**Table S1** Age-adjusted hazard ratios for pre-diagnostic syndecan-1, syndecan-4, breast cancer-specific survival, and overall survival among women diagnosed with breast cancer.

|  | **Cases/total**  **(n)** | **HR^a^ (95% CI)** |
| --- | --- | --- |
| **Syndecan-1** |  |  |
| **BCSS** |  |  |
| Continuous, per 1 SD^b^ increase | 20/140 | 0.64 (0.17-2.46) |
| Quartiles |  |  |
| Q1: ≤62.67 ng/mL | 5/32 | 1.00 (Reference) |
| Q2: 62.67- 87.60 ng/mL | 4/38 | 0.62 (0.16-2.30) |
| Q3: 87.61-132.00 ng/mL | 9/38 | 1.59 (0.51-4.89) |
| Q4: ≥132.00 ng/mL | 2/32 | 0.30 (0.06-1.60) |
| **OS** |  |  |
| Continuous, per 1 SD^b^ increase | 57/140 | 0.53 (0.20-1.41) |
| Q1: ≤62.67 ng/mL | 11/32 | 1.00 (Reference) |
| Q2: 62.67- 87.60 ng/mL | 16/38 | 1.15 (0.53-2.49) |
| Q3: 87.61-132.00 ng/mL | 20/38 | 1.30 (0.62-2.72) |
| Q4: ≥132.00 ng/mL | 10/32 | 0.59 (0.25-1.42) |
| **Syndecan-4** |  |  |
| **BCSS** |  |  |
| Continuous, per 1 SD^c^ increase | 24/158 | 1.28 (0.91-1.82) |
| Quartiles |  |  |
| Q1: ≤16.08 ng/mL | 2/22 | 1.00 (Reference) |
| Q2: 16.08-19.60 ng/mL | 5/45 | 1.22 (0.24-6.36) |
| Q3: 19.61-23.70 ng/mL | 9/44 | 2.39 (0.51-11.10) |
| Q4: ≥23.70 ng/mL | 8/47 | 2.19 (0.45-10.03) |
| **OS** |  |  |
| Continuous, per 1 SD^c^ increase | 67/158 | 1.17 (0.93-1.46) |
| Quartiles |  |  |
| Q1: ≤16.08 ng/mL | 9/22 | 1.00 (Reference) |
| Q2: 16.08-19.60 ng/mL | 16/45 | 1.03 (0.45-2.38) |
| Q3: 19.61-23.70 ng/mL | 20/44 | 1.32 (0.59-2.93) |
| Q4: ≥23.70 ng/mL | 22/47 | 1.48 (0.67-3.24) |

^a^Age-adjusted (continuous)

^b^ SD=197.81 ng/ml

^c^ SD=5.75 ng/ml

Abbrevations: HR, hazard ratio; CI, confidence interval; n, number of participants; SD, standard deviation; BCSS, breast cancer-specific survival; OS, overall survival

1. Solbu MD, Kolset SO, Jenssen TG, Wilsgaard T, Løchen M-L, Mathiesen EB, et al. Gender differences in the association of syndecan-4 with myocardial infarction: The population-based Tromsø Study. Atherosclerosis. 2018;278:166-73.
